# Childhood Obesity and Overweight Are Associated with Higher Risk of Perceived Stress and Poor Sleep Quality: A Cross-Sectional Study in Children Aged 6–9 Years

**DOI:** 10.3390/metabo15060345

**Published:** 2025-05-22

**Authors:** Maria Mentzelou, Aikaterini Louka, Theophanis Vorvolakos, Maria G. Kapetanou, Aspasia Seradri, George Antasouras, Christos Kontogiorgis, Georgia-Eirini Deligiannidou, Maria Chrysafi, Constantinos Giaginis

**Affiliations:** 1 Department of Food Science and Nutrition, School of the Environment, University of the Aegean, 81400 Myrina, Lemnos, Greece; maria.mentzelou@hotmail.com (M.M.); loukathy612@gmail.com (A.L.); mariakaptain@yahoo.gr (M.G.K.); g.antasouras@gmail.com (G.A.); m.chrisafi3@gmail.com (M.C.); 2 Department of Psychiatry, School of Medicine, Democritus University of Thrace, 68100 Alexandroupolis, Greece; tvorvola@med.duth.gr; 3 Department of Child and Adolescent Psychiatry, School of Medicine, Democritus University of Thrace, 68100 Alexandroupolis, Greece; aserdari@yahoo.com; 4 Laboratory of Hygiene and Environmental Protection, Department of Medicine, School of Health Sciences, Democritus University of Thrace, 68100 Alexandroupolis, Greece; ckontogi@med.duth.gr (C.K.); deligiannidoueirini@yahoo.gr (G.-E.D.); 5 Department of Nutritional Sciences and Dietetics, School of Health Sciences, International Hellenic University, 57001 Thessaloniki, Greece

**Keywords:** childhood obesity, perceived stress, sleep quality, primary school children, sociodemographics, perinatal outcomes, breastfeeding practices, lifestyle factors

## Abstract

**Background/Objectives:** The number of children with overweight and obesity is gradually increasing worldwide. This is an emergent public health problem as overweight and obesity persist through the next stages of human life, being associated with high risk of morbidity and mortality. In this respect, the purpose of the current cross-sectional survey is to explore whether the overweight/obesity of children aged 6–9 years may be related to the risk of developing perceived stress and poor sleep quality symptoms. **Methods:** This study recruited 4350 primary school children from diverse Greek rural and urban regions. The mothers of the enrolled children completed relevant questionnaires on children and maternal sociodemographics, anthropometric parameters, perinatal outcomes, breastfeeding practices, and lifestyle factors. The enrolled mothers also completed the Perceived Stress Scale (PSS) and the Pittsburgh Sleep Quality Index (PSQI) to evaluate the perceived stress and sleep quality of their matched children, respectively. **Results**: Our analysis reveals independently significant associations between diverse factors and children’s overweight/obesity such as child’s gender, family economic status, maternal gestational weight gain, childbirth weight, kind of delivery, exclusive breastfeeding, and children physical activity. Childhood overweight and obesity were independently associated with a more than three-fold higher risk of perceived stress and a more than two-fold higher likelihood of poor sleep quality after adjustment for multiple confounding factors. **Conclusions:** Obesity and overweight are related to enhanced risk of perceived stress and poor sleep quality in children aged 6–9 years. Public policies and dietary counseling interventions should be applied to inform new mothers about the harmful effects of childhood overweight/obesity and to direct them to adopt healthy dietary practices for their children.

## 1. Introduction

In the last few years, childhood overweight and obesity have become an emergent public health problem worldwide, which has currently reached epidemic proportions [1]. It has recently been considered a chronic inflammatory process that may lead to persistent obesity in adolescence and adulthood [2]. Several risks and etiological factors may trigger childhood obesity, such as endocrine, genetic, environmental, societal, and sedentary behavior factors [3]. Childhood overweight/obesity is not equally distributed across countries and populations in the European Union, with over 398,000 children at the age of 6–9 years facing severe obesity in 2019 [4,5].

In Greece, the prevalence of childhood overweight/obesity approached approximately above the 25% of the childhood population with increasing trends expected in the next few years [6,7]. A Greco study reported even higher overweight and obesity prevalence rates, which were estimated to be 29.5% and 13.1%, respectively, among boys aged 10–12 years, and 29.5% and 9.0%, respectively, among girls at the same age [8]. Childhood overweight/obesity is highly associated with enhanced probability of diverse chronic diseases like metabolic disorders, cardiovascular diseases, cancer, and mental disorders [9,10]. Its increasing rates are associated with short- and long-term health and socioeconomical expenses of non-communicable diseases, including mental disorders [11]. The extensive intake of ultra-processed foods and junk foods (e.g., fast foods, sugary snacks, energy-dense drinks, etc.) in childhood has also been related to disturbed diverse metabolic biomarkers [12].

Both short-term and long-term psychosocial and mental health disorders during childhood and adolescence were a significant public health problem before the COVID-19 pandemic [13,14]. Moreover, mental health challenges including anxiety, stress, depression, suicide attempts, sleep alterations, and eating disorders among children and adolescents increased during COVID-19 pandemic [15,16]. In Greece, several mental health conditions have been raised due to extended socioeconomic backdrops, the COVID-19 pandemic, and the limited capacity of the healthcare system [17]. Mental health disorders, including stress, can affect Greek children’s and adolescents’ lives with a prevalence of 24.6% of disability-adjusted life years lost across all diseases concerning the age group of 5–14 years [17]. Financial crisis exerted a considerable effect on mortality due to mental diseases, particularly in females and older adults [18].

Furthermore, about 11% of adolescents suffer from mental health symptoms away from the cut-off for professional assessment, and the COVID-19 lockdown could have additionally diminished these figures [19]. Among mental disorders, perceived stress in children has been associated with harmful health outcomes during the lifespan [20]. Amongst the diverse sources of stress, perceived stress and its emotive reactions considerably influence well-being [21]. Several studies have revealed elevated levels of stress among girls despite their age, as well as an association of perceived stress with heart rate variables. An enhanced perceived stress level has also been related to reduced socioeconomic level, which considerably affected the capability of families to cope with stress [22].

Short sleep duration influences about 25–30% of adults and 35% of children and adolescents in spite of the guidelines for regular and sufficient sleep to provide beneficial health [23]. Adequate sleep quality is highly necessary for great health due to its direct act on neuronal, cognitive, immune, and appropriate growth/development [24]. Sleep is a spontaneous and modifiable resting condition in mammals, which is crucial for improving energy management or distribution, modulating core molecular and cellular procedures, and enhancing nerve system functionality [25].

In the last few decades, the mean nightly sleep interval has reduced, with sleep disturbances like insomnia continuing to elevate in incidence [26]. Among others, sleep disturbances have been related to elevated stress levels, which may enhance the probability of loneliness, negative affect, lethargy, and napping functions [27]. The incidence of sleep disturbances was 35.7% concerning the general population throughout the COVID-19 confinement [28]. An even higher incidence of sleep disturbances of 45.96% among children and adolescents throughout the COVID-19 lockdown was recorded [29]. The pooled prevalence of any sleep disturbance was additionally increased to 54% at the end of COVID-19 lockdown in childhood and adolescence in the presence or absence of neurobehavioral and psychiatric diseases [30].

Sleep and stress are directly related, at multiple levels, presenting a bidirectional association [31]. A potential relationship between sleep health and neuroendocrine, immune, metabolic, and cardiovascular biomarkers in healthy children aged 0–12 years was noted [32]. Long sleep duration, instead of short sleep duration, was also considerably related to a lower incidence of metabolic syndrome among adolescents and children [33,34]. The COVID-19 quarantine also brought about significant changes in sleeping habits, which continued after its termination in Greece [35].

In addition, stress can induce insulin resistance, increasing the probability of obesity by deregulating blood glucose levels and adipose tissue storage and affecting the gut microbiota, which can potentially affect chronic inflammation and metabolic processes linked to obesity [36,37]. In this respect, both diabetes and obesity seem to be related to a higher risk of developing several types of cancer, while chronic stress has been considered to substantially increase this association [38]. Moreover, stress exposure in children and adolescents can promote multiple biological and behavioral modifications, which can influence the multifactorial obesity pathogenesis, while COVID-19 associated-stress presented the most current example of an adverse effect on body weight growth in childhood and adolescence [39].

Emerging evidence highlights a bidirectional relationship between obesity and sleep disorders with each worsening the other in a complex interaction of behavioral, physiological, and hormonal mechanisms [40]. Sleep deprivation and poor sleep quality appear to contribute to energy imbalance through the dysregulation of appetite hormones such as leptin and ghrelin, increasing caloric intake, and reducing physical activity [41,42]. Conversely, sleep disorders like obstructive sleep apnea syndrome, insomnia, and restless leg syndrome have been shown to substantially occur more frequently in individuals with obesity [43]. Inadequate sleep and circadian disturbance can also lead people to metabolic disorders, promoting weight gain [34,44]. Shortened sleep duration and later bedtimes (sleep timing) can elevate the risk of weight gain in children and adolescents, and higher body mass index (BMI) values have been associated with shorter sleep duration from adolescence to adulthood [45].

Although there are several studies examining the relationships of overweight/obesity on stress conditions and sleep quality, these studies are mainly focused on adulthood or adolescent and not on children. Moreover, the currently existing clinical evidence concerning the above relationships remains contradictory, while clinical studies separately examining the associations of childhood overweight/obesity with perceived stress and the associations of childhood overweight/obesity with sleep quality remain scarce. In this respect, the present study aims to simultaneously assess whether overweight/obesity could be related to an elevated probability of perceived stress and inadequate sleep quality in children at the age of 6–9 years after adjustment for multiple sociodemographics, anthropometric parameters, perinatal outcomes, breastfeeding practices, and physical activity.

## 2. Methods

### 2.1. Study Population

**Figure 1:**
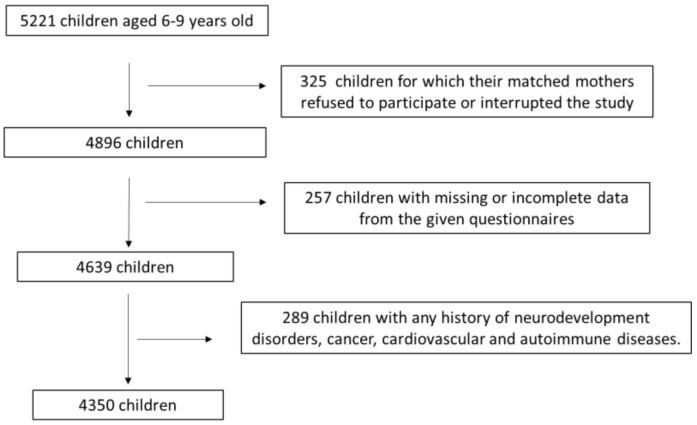
Flow chart diagram for study enrolment.

This is a cross-sectional study that initially involved 5221 primary school children at the age of 6–9 years old and their paired mothers who were enrolled from 10 urban and rural geographical regions of Greece, containing Athens, Thessaloniki, Larissa, Patra, Alexandroupolis, Kalamata, Ioannina, Crete, and South and North Aegean. Recruitment to this study was randomly achieved between May 2018 and September 2022. The majority of mothers were assigned during their visits to primary education schools. This study included, during the first admission, children aged 6–9 years old without any chronic diseases like cardiovascular disease, metabolic illnesses, and tumor malignancies. In total, 4350 children and their paired mothers ultimately joined in the survey analysis utilizing the above inclusion and exclusion criteria, resulting in a final response rate of 83.3%. A flow chart diagram of the survey assignment is depicted in Figure 1. Convenience sampling was performed, which is a non-probability sampling method that is the most applicable and widely used method in relevant research. In this method, we enrolled subjects according to their availability and accessibility. This method is quick, inexpensive, and convenient. Sample size calculation was estimated for a population size of 100,000 with a confidence level of 95% and a margin of error of 5%.

All participants’ data were kept severely private. The enrolled mothers of their matched children were informed regarding the purposes of the survey, and they signed a consent form, verifying their agreement for probable publishment of their confidential data namelessly. The survey was authorized by the Ethical Agency of the University of the Aegean (ethical approved protocol: no 12/14.5.2016) and was in accordance with the World Health Organization (52nd WMA General Assembly, Edinburgh, Scotland, 2000). Sample size assessment was estimated utilizing the PS: Power and Sample Size calculator program. The sample was randomly enrolled. The estimation of the power of the study sample size was a power of 87.8%.

### 2.2. Study Design

#### 2.2.1. Sociodemographic Parameters

Throughout this study, relevant questionnaires were utilized for evaluating the sociodemographic parameters of the enrolled children such as children age, gender (boys vs. girls), nationality (Greek vs. others), and type of residence (urban vs. rural) through one-to-one interviews between the assigned matched mothers with qualified nutritionists or dietitians to minimize recall biases [46,47]. Other nationalities besides Greeks were included in this study only in the case they spoke and understood the Greek language. The mothers’ education status was categorized into 3 classes: (a) primary and (b) secondary education, as well as (c) university studies. Financial status was grouped according to the annual family income as follows: 0: < EUR 5000; 1: EUR 5000–10,000; 2: EUR 10,000–15,000; 3: EUR 15,000–20,000; 4: EUR 20,000–25,000; 5: > EUR 25,000. Financial status was further categorized as low for family annual income ≤ EUR 10,000, medium for annual income > EUR 10,000 and ≤ EUR 20,000, and high for annual income > EUR 20,000 [46,47]. Mothers’ sociodemographic parameters like marital and employment status, smoking habits, and parity were also collected by one-to-one interviews of the enrolled women with qualified personnel to minimize recall biases.

#### 2.2.2. Perinatal Outcomes

Perinatal outcomes, including maternal gestational weight gain (GWG), pre-pregnancy BMI, childbirth body weight, and the type of childbirth (vaginal or cesarean section), were recovered from the women’ medical documents. Based on the Institute of Medicine’s (IOM) guidelines, the proposed GWG for underweight women pre-pregnancy (BMI < 18.5 kg/m^2^) was between 12.5 and 18.0 kg; for women with normal weight (BMI: 18.5–24.9 kg/m^2^), between 11.6 and 16.0 kg; for women with overweight (BMI: 25.0–29.9 kg/m^2^), between 7.0 and 11.5 kg; and for women with obesity (BMI ≥ 30.0 kg/m^2^), between 5 and 9 kg [48]. The enrolled women were grouped based on the above criteria into three groups: women with a lower GWG compared to that suggested, (b) women with a normal GWG, and (c) women with excessive GWG. Childbirth weight was also retrieved from maternal medical documents, being categorized as low (<2500 g), normal (2500–4000 g), and high (>4000 g), as recommended by the appropriate literature [49]. Mother’s and children’s weights were measured using a Seca scale [Seca, Hanover, MD], without shoes, to the nearest 100 g, and height was measured using a portable stadiometer (GIMA Stadiometer 27335, Athens, Greece) with no shoes on, to the nearest 0.1 cm. To estimate maternal pre-pregnancy BMI, anthropometric parameters throughout the initial gestational weeks were determined during a visit to their individual gynecologists or to public or private hospitals. Both weight and height parameters for women throughout the initial gestational weeks were retrieved from their medical documents; therefore, they had been measured and were not self-reported.

#### 2.2.3. Anthropometric Parameters

Children anthropometric parameters such as body weight, and height were measured throughout the interval of study by qualified personnel. Body weight was determined using the same electronic scale, and height was determined utilizing a portable stadiometer [46,47]. The body weight was measured near to the closest 100 g, and the height was determined near to the closest 0.50 cm. The International Obesity Task Force (IOTF) recommendations were used to classify the assigned children with normal weight, overweight, or obesity [50,51].

#### 2.2.4. Breastfeeding Practices

Mothers were asked whether they applied exclusive breastfeeding for a minimum interval of 4 months. To reduce recall biases, the mothers were asked for exclusive breastfeeding for a minimum period of 4 months since most of them were advised to progressively familiarize pulp foods to the nourishing strategies of their children at the end of the 4th month and the opening of the 5th month, and thus, they are capable of recalling more exactly this time point, making their responses more consistent. In contrast, mothers breastfeeding for shorter intervals were not capable of answering with sufficient confidence concerning the exact breastfeeding period [52,53].

#### 2.2.5. Children’s Physical Activity

The physical activity of children was estimated utilizing the International Physical Activity Questionnaire (IPAQ) Long Form in which the mothers reported the interval of any physical activity of their children throughout a typical week. This self-reported questionnaire is used globally and evaluates total physical activity regarding the last 7 days, to categorize it as low, moderate, or high [54]. IPAQ instruments have comprehensively been tested and represent sufficient reliability and validity, at least as effective as other self-reported PAQs [54]. The IPAQ for the children was completed by their matched mothers.

#### 2.2.6. Children’s Perceived Stress and Sleep Quality

The Perceived Stress Scale (PSS) was applied for assessing the stress of the assigned children [55]. This is a 10-item questionnaire invented to estimate the self-reported levels of children’s stress by evaluating thoughts and emotions throughout the previous few months. Items were formed to determine whether the assigned children estimate their lives as unpredictable, uncontrollable, and overloaded. The PSS has been characterized with a validity of high quality in current research [56]. The mothers are recommended to estimate how frequently their children experienced a specific feeling in the last month. Each question ranged from 0 (never) to 5 (very often). The total score, derived by the sum of questions, may range from 0 to 40, and perceived stress is higher as the score increases. The PSS score is classified into 3 categories low (score: 0–3), moderate (score: 14–26), and high (score: 27–40) perceived stress [56]. In our study, the PSS for children was completed by their matched mothers. The PSS measures the psychological stress associated with many demographics. These include age, education, employment status, gender, and income. Perceived stress is an indicator for subjects because perception varies a great deal.

Sleep quality was estimated utilizing the Pittsburgh Sleep Quality Index (PSQI), which involves 19 items ranged on a 4-point scale (0–3) and classified in 7 components (sleep quality, sleep latency, sleep period, habitual sleep effectiveness, sleep impairment, use of sleeping medicines, and daytime disfunction). The item scores in each component were added and transformed to component scores ranging from 0 (better) to 3 (worse) according to the relevant guidelines [57]. Total PSQI scores were projected as the sum of 7 component scores ranging from 0 to 21, where a higher score indicates an adverse circumstance. A whole overall PSQI score of <5 represents adequate sleep quality [57]. All overhead questionnaires were carried out by qualified physicians (e.g., medical and nursing personnel) and nutritionists and dietitians throughout face-to-face interviews with the assigned mothers. In our study, the PSQI for children was completed by their matched mothers. The PSQI is commonly used in both clinical and research settings to evaluate various aspects of sleep. It is a valuable tool for assessing sleep quality as it captures multiple dimensions of sleep, including both subjective experiences and objective parameters. It allows researchers and healthcare providers alike to obtain a comprehensive understanding of an individual’s sleep patterns and disturbances and inform treatment decisions and interventions for sleep disorders.

### 2.3. Statistical Analysis

Student’s *t*-test was used for the continuous variables presenting a normal distribution. The Kolmogorov–Smirnov test was applied for checking the distribution normality of the continuous variables. The chi-square test was used for categorical variables. Mean value ± Standard Deviation (SD) was utilized for the quantitative variables that exhibited a normal distribution. The qualitative variables are expressed as absolute or relative frequencies. Multivariate binary logistic regression analysis was applied for assessing whether children with overweight/obesity may be independently associated with sociodemographics, anthropometric parameters, perinatal outcomes, breastfeeding practices, lifestyle factors, perceived stress, and sleep quality after adjustment for probable confounding factors. The Statistica 10.0 software, Europe, was applied for the statistical analysis (Informer Technologies, Inc., Hamburg, Germany).

## 3. Results

In Table S1 (Supplementary Material), the descriptive statistics of the survey participants are depicted. The mean age of children was 7.1 ± 1.2 years old with a male–female ration almost equal to the unit. In total, 95.7% of the children had a Greek nationality, and 65.6% of them lived in urban areas of Greece. In total, 30.3% of their mothers had a low educational level; 42.6%, a moderate level; and 27.1%, a high level. In total, 42.0% of children had a low family economic status; 37.5%, a moderate status; and 20.5% a high status. In total, 74.2% of mothers were not smokers, 68.5% were employed, 68.2% were married, and 64.7% were nulliparous. The mean age of mothers was 35.4 ± 5.1 years.

Based on pre-pregnancy BMI, 17.0% of the enrolled mothers were overweight, and 4.6% were obese. According to IOM guidelines, 14.7% of mothers were characterized by low GWG and 38.9% with excessive GWG. In total, 7.9% of children had a low birth weight and 10.4% had a high birth weight. In total, 56.3% of mothers gave birth by cesarean section delivery, while exclusive breastfeeding was adopted by 50.2% of mothers.

Only 12.8% of children showed high physical activity, while 39.5% and 47.5% had moderate and low physical activity, respectively. In total, 62.2% of children showed low perceived stress; 32.6%, moderate stress; and 5.2%, high stress. In total, 25.8% of children had inadequate sleep quality and 74.2% had adequate sleep quality.

In cross-tabulation, the potential associations of the children’s BMI with the collected variables were examined (Table 1). Overweight/obesity was significantly more frequently observed in girls than boys (*p* ˂ 0.0001). A lower maternal educational level was significantly more often seen in children affected by overweight or obesity compared to those presenting normal weight (*p* = 0.0045). Family economic status was significantly reduced in children affected by overweight or obesity compared to those presenting normal weight (*p* = 0.0031). Mothers who were regular smokers were significantly and more frequently seen in children with overweight and obesity than non-smoker mothers (*p* ˂ 0.0001). Children with overweight and obesity were significantly observed more frequently in multiparous compared to nulliparous mothers (*p* ˂ 0.0001).

Children’s BMI was positively associated with maternal pre-pregnancy BMI status (*p* ˂ 0.0001). Excessive maternal GWG was more frequently and significantly associated with children with overweight and obesity (*p* = 0.0002). High childbirth weight was significantly observed more often in children presenting overweight or obesity than those with normal weight (*p* = 0.0001). Children born by cesarean section were considerably more often overweight or obese compared to children born by vaginal delivery (*p* ˂ 0.0001). Children who were not exclusively breastfed were significantly associated with overweight or obesity compared to children who were exclusively breastfed (*p* ˂ 0.0001).

Children’s low physical activity was significantly associated with overweight and obesity compared to those presenting moderate or high physical activity (*p* ˂ 0.0001). Overweight and obesity were significantly observed more frequently in children presenting perceived stress symptoms (*p* ˂ 0.0001). Overweight and obesity were also considerably more frequently found in children presenting inadequate sleep quality (*p* ˂ 0.0001).

In multivariate binary logistic regression analysis, children’s gender, maternal GWG, childbirth weight, kind of delivery, breastfeeding practices, children’s physical activity, perceived stress, and sleep quality were significantly and independently associated with children’s BMI (Table 2, *p* ˂ 0.05). In fact, girls had a 38% higher prevalence of overweight or obesity than boys (*p* = 0.0341). Children with low family economic status showed a 39% higher probability of being overweight or obese than those with moderate or high family economic status (*p* = 0.0293).

Children whose mothers had excessive GWG exhibited an 88% greater probability of being overweight or obese at the age of 6–9 years (*p* = 0.0291). Children with low or high birth weight had a 68% greater risk of presenting overweight or obesity compared to those presenting normal birth weight (*p* = 0.0278). Children born by cesarean section showed a two-fold elevated risk of developing overweight or obesity compared to those born by vaginal delivery (*p* = 0.0248). Children who did not receive exclusive breastfeeding as well as those presenting low physical activity had a more than two-fold higher probability of being overweight or obese (*p* = 0.0045 and *p* = 0.0087, respectively).

Children affected by overweight or obesity had a more than three-fold greater risk of developing perceived stress (*p* = 0.0013) than children with normal weight. Children affected by overweight or obesity also exhibited a more than two-fold greater probability of developing inadequate sleep quality (*p* = 0.0013) than children with normal weight (*p* = 0.0052).

## 4. Discussion

This is the first cross-sectional survey in Greece and the world investigating whether childhood overweight/obesity at the age of 6–9 years may be related to the likelihood of perceived stress and poor sleep quality symptoms. The present study provides evidence that childhood overweight/obesity was independently, more frequently observed in female children with low family economic status, whose mothers had excessive GWG and a cesarean delivery. Moreover, childhood overweight/obesity was independently more often observed in children not adopting exclusive breastfeeding in the first months of their life as well as in those with low levels of physical activity. Notably, childhood overweight/obesity was independently associated with a more than three-fold higher risk of perceived stress and a more than two-fold higher likelihood of poor sleep quality after adjustment for multiple confounding factors. Therefore, we can suggest that obesity and overweight in children may lead to stress and sleep disorders. In contrast, we cannot support that stress and sleep disorders could be the cause of childhood obesity/overweight due to the cross-sectional design of our study.

There are several studies that support that perceived stress may exert a crucial effect on metabolic diseases, including obesity [58,59,60,61,62,63,64,65]. However, almost all current studies have focused on adolescence and adulthood [58,59,60,61,62,63,64,65], rendering our study as one of the first studies worldwide that assessed the association of perceived stress with overweight/obesity in primary school children at the age of 6–9 years. Considering the currently existing studies, Park et al. showed that perceived stress levels, age, and diastolic blood pressure exert a substantial impact on the obesity rate in adult males [58]. Moreover, they found that the impact of perceived stress level on obesity and hypertension rates had a lower prevalence in the elderly population compared to the adults [58]. In this respect, Roy et al. found that perceived stress was positively and considerably related to eating patterns and BMI, while physical activity was notably inversely related to the incidence of overweight/obesity and high stress in urban adolescents [59]. In a previous cross-sectional study, perceived stress was positively related to uncontrolled eating and emotional eating, which may lead to overweight/obesity. Notably, stress was positively related to severe obesity in low-income women independently of eating behaviors and diet quality [60]. Moreover, elevated perceived stress levels were related to reduced levels of eating awareness, physical activity, and walking in adults. Amongst participants with decreased levels of eating awareness, elevated perceived stress levels were related to less servings of fruit and vegetables and higher intake of fast-food meals, which may increase the rates of obesity [61].

Furthermore, Farag et al. showed that psychological stress can predict a considerable portion of hypothalamic–pituitary–adrenal (HPA) axis functioning in adults. Moreover, in overweight women, perceived stress and waist circumference were of approximately equal importance in predicting adrenal cortisol secretion [62]. Another study reported that amongst children with overweight or obesity, parent-perceived stress was related to fast-food intake and physical activity. Parent-perceived stress was also related to childhood BMI concerning low-income households and non-Hispanic black children [63]. Moreover, a meta-analysis showed a substantial association among stress, BMI, waist circumference, and serum triglyceride levels in patients with metabolic syndrome. In this respect, additional analysis confirmed the impact of stress on serum HDL and diastolic blood pressure [64]. Accordingly, in a recent study designed by our research group, it was shown that young adults with medium or elevated perceived stress levels exhibited a more than two-fold incidence of overweight/obesity than young adults presenting reduced perceived stress levels [65].

In accordance with our study, a small pilot survey conducted on 37 primary school children indicated that most of the children with obesity, 12 out of 19, exhibited inadequate sleep quality, whereas most of the children without obesity, 15 out of 18, presented adequate sleep quality. Moreover, primary school children presenting inadequate sleep quality had 8.6 greater times to develop obesity compared to those exhibiting adequate sleep quality [66]. In a sample of 125 children/adolescents (mean age = 13.06, 9–17-year-olds), the video-game play period in the four-hour window prior to bedtime, characteristic video-game session period, sweet drink intake while playing video games, and low sleep quality showed aversive relations to abdominal adiposity [67]. Moreover, in a cross-sectional study of 416 adolescents at the age of 12–18 years, participants with obesity had lower sleep quality and a longer period of social jet lag than participants with normal weight [68]. However, in a case–control survey, performed in children aged 7–13 years, no considerable relationship of sleep quality/duration with overweight/obesity was noted [69]. Another cross-sectional study in adolescents supported that reduced total sleep duration, weekend sleep duration, and night sleep duration were substantially related to overweight or obesity in Bangladeshi adolescents [70].

In addition, Hur et al. found that obesity was related to elevated rates of inadequate sleep quality in women, but not in men. Notably, by adjusting for several covariates, women presenting inadequate sleep quality exhibited an elevated probability of obesity compared to women presenting adequate sleep quality [71]. Accordingly, another cross-sectional study reported that young adults who had poor sleep quality were two-fold more probable to develop overweight or obesity than those who had adequate sleep quality after adjustment for several confounding factors like age, gender, nationality, and acculturative stress [72]. In another study conducted on older farmers and their spouses revealed that BMI was positively associated with sleep apnea symptoms, and participants presenting difficulty falling asleep were more probable to exhibit inadequate sleep quality [73]. Accordingly, in a recent study designed by our research group, it was shown that young adults presenting poor sleep quality exhibited a more than two-fold incidence of overweight/obesity compared to young adults presenting sufficient sleep quality [65]. In agreement with our study, a previous cross-sectional study in Greece also showed a negative relationship of mean sleep duration on weekdays and weekends with the risk of being overweight/obese of children aged 10–12 years [74].

Our results reveal a greater incidence of overweight or obesity amongst female children than their male counterparts. This finding agrees with the currently available literature that has indicated a greater overall obesity incidence in female (40%) compared to male (35%) adults based on the National Health and Nutrition Examination Survey (NHANES) performed between 2005 and 2014 [75], as well as with our previous recent study in young adults [65]. We also found that childhood overweight/obesity was more frequently observed in children with low family economic status, which may be ascribed to the fact that low-income families have limited access to healthy dietary patterns, which are usually more expensive [76,77]. The present study also shows that childhood overweight/obesity was more often in children whose mothers had excessive GWG and cesarean delivery. The above evidence is in accordance with previous studies highlighting that excessive maternal GWG [78,79] and cesarean delivery [80,81,82] may significantly increase the probability of developing overweight and obesity during the initial steps of children life. Children not adopting exclusive breastfeeding in the first months of their life as well as in those with low levels of physical activity during the initial stages of their life also had a substantial increased risk of developing overweight/obesity, as previously reported by our research group and others [46,53,83,84,85].

The present study has several strengths as it was performed in diverse Greek areas, containing both urban and rural settings and including a large sample size of children and their matched mothers. The above facts enhance the generalizability of our results and support evidence that the study population sample is quite representative of primary school children in Greece. In addition, the usage of real BMI measures instead of self-reported data guaranteed the precise classification of the enrolled children into overweight or obese categories, reducing recall biases. In addition, children’s sociodemographics, perinatal outcomes, and breastfeeding practices were estimated through one-to-one interviews between the enrolled mothers with qualified personnel or were retrieved from their medical records, which further minimized self-reported recall biases. Lastly, physical activity, perceived stress, and sleep quality were determined using well-known and certified questionnaires via one-to-one interviews that further increase the validity of mothers’ responses, and the evaluation of the under-study childhood obesity risk factors.

This survey is characterized by certain limitations that need to be taken into careful account. The cross-sectional design of our survey did not permit us to conclusively obtain causality effects of perceived stress and sleep quality with childhood overweight/obesity. In this respect, performing longitudinal studies could permit the exploration of causality associations amongst the above factors in primary school children. We also conducted comprehensive evaluations encompassing various sociodemographics, anthropometrics, perinatal outcomes, breastfeeding practices, and lifestyle factors, underscoring the depth of our research approach. In this respect, despite our efforts to minimize recall bias through face-to-face meetings, the dependence on self-reported data does not avoid the presence of potential confounders. However, such data are frequently utilized, and they are commonly consistent in cross-sectional surveys. In any case, we should highlight that the self-reported data from questionnaires could result in potential biases. In addition, adjusting for multiple confounding factors could not cover all related variables like mental health diseases such as depression, anxiety, and multiple eating disorders amongst children (e.g., anorexia nervosa, bulimia nervosa, emotional eating, etc.), which may have affected our results as residual confounders. The dietary habits of the children could be another confounding factor for which we will design a new research in the future. Moreover, we used the IPAQ to assess the physical activity levels of the enrolled children; it has been validated for adolescents and adults (age range: 15–69 years) but not for children. However, it should be noted that no questionnaires for children currently exist with conclusive evidence for both acceptable validity and reliability, partly due to the low methodological quality of the currently available studies [86]. Taking into consideration these limitations in future studies will be essential for improving our knowledge of whether childhood overweight/obesity may affect childhood health outcomes, contributing to the current knowledge and carry out more effective obesity prevention strategies at the first stage of human life.

## 5. Conclusions

The present study provided substantial evidence that overweight/obesity may increase the risk of developing perceived stress and poor sleep quality in primary school children. Childhood overweight/obesity was also more frequently observed in female children with low family economic status and physical activity levels, whose mothers had excessive GWG and cesarean delivery and did not adopt exclusive breastfeeding. The above findings emphasize the significance of a multifaceted approach to body weight management in this childhood population. These findings also advocate for public health strategies in primary schools to confront perceived stress and poor sleep quality in this target population group. Primary school programs and initiatives as well as public health policies can play a critical role in promoting healthy lifestyles and well-being in children aged 6-9 years by systematically advising their mothers. Moreover, future prospective studies should explore causal relationships and examine the long-term effects of perceived stress and poor sleep quality in the body weight management of this children group.

## Figures and Tables

**Table 1:** Associations of childhood BMI with sociodemographics, anthropometric parameters, perinatal outcomes, breastfeeding practices, lifestyle factors, perceived stress, and sleep quality.

Characteristics (n = 4350)	Childhood BMI			
	Normal Weight 3281 (75.4%)	Overweight 728 (16.7%)	Obesity 341 (7.9%)	*p*-Value
Childhood age (mean ± SD; years)	7.52 ± 1.03	7.50 ± 1.07	7.53 ± 1.08	*p* = 0.3781
**Gender (n, %)**				*p* ˂ 0.0001
Male	1738 (53.0%)	275 (37.8%)	153 (44.9%)	
Female	1543 (47.0%)	453 (62.2%)	188 (55.1%)	
**Nationality (n, %)**				*p* = 0.1399
Greek	3152 (96.1%)	690 (94.8%)	322 (94.4%)	
Other	129 (3.9%)	38 (5.2%)	19 (5.6%)	
**Type of residence (n, %)**				*p* = 0.6768
Urban	2381 (72.6%)	540 (74.2%)	239 (70.1%)	
Rural	900 (27.4%)	188 (25.8%)	102 (29.9%)	
**Maternal educational level (n, %)**				*p* = 0.0045
Low	958 (29.2%)	231 (31.7%)	127 (37.2%)	
Moderate	1402 (42.7%)	306 (42.0%)	146 (42.8%)	
High	921 (21.2%)	191 (6.3%)	68 (20.0%)	
**Family economic status (n, %)**				*p* = 0.0031
Low	1354 (41.3%)	313 (43.0%)	160 (46.9%)	
Moderate	1227 (37.4%)	297 (40.8%)	107 (31.4%)	
High	700 (21.3%)	118 (16.2%)	74 (21.7%)	
**Maternal age (mean ± SD; years)**	35.8 ± 6.4	35.2 ± 6.2	35.6 ± 6.7	*p* = 0.7339
**Maternal smoking habits (n, %)**				*p* ˂ 0.0001
Non-smokers	2485 (75.7%)	533 (73.2%)	209 (61.3%)	
Regular smokers	796 (24.3%)	195 (26.8%)	132 (38.7%)	
**Employment status (n, %)**				*p* = 0.2596
Employed	2268 (69.1%)	487 (66.9%)	224 (65.7%)	
Unemployed	1013 (23.3%)	241 (33.1%)	117 (34.3%)	
**Marital status (n, %)**				*p* = 0.7669
Married	2230 (68.0%)	505 (69.4%)	233 (68.3%)	
Divorced	1051 (32.0%)	223 (30.6%)	108 (31.7%)	
**Parity (n, %)**				*p* ˂ 0.0001
Nulliparity	2185 (66.6%)	414 (56.9%)	216 (63.3%)	
Multiparity	1096 (33.4%)	314 (43.1%)	125 (36.7%)	
**Maternal pre-pregnancy BMI status (n, %)**				*p* ˂ 0.0001
Underweight	115 (3.5%)	11 (1.5%)	0 (0.0%)	
Normal weight	2496 (76.1%)	570 (78.3%)	219 (64.2%)	
Overweight	560 (17.1%)	90 (12.4%)	91 (26.7%)	
Obese	110 (3.3%)	57 (7.8%)	31 (9.1%)	
**Maternal gestational weight gain (n, %)**				*p* = 0.0001
Low	466 (14.2%)	144 (19.8%)	31 (9.1%)	
Normal	1558 (47.5%)	308 (42.3%)	156 (45.7%)	
Excessive	1257 (38.3%)	276 (37.9%)	154 (45.2%)	
**Childbirth weight (n, %)**				*p* ˂ 0.0001
Low birth weight (<2500 gr)	288	50	6	
Normal birth weight (2500–4000 gr)	2708	569	276	
High birth weight (>4000 gr)	285	109	59	
**Kind of delivery (n, %)**				*p* ˂ 0.0001
Vaginal	1568 (47.8%)	264 (36.3%)	68 (19.9%)	
Cesarean section	1713 (52.2%)	464 (63.7%)	273 (80.1%)	
**Exclusive breastfeeding (n, %)**				*p* ˂ 0.0001
No	1486 (45.3%)	424 (58.2%)	257 (75.4%)	
Yes	1795 (54.7%)	304 (41.8%)	84 (24.6%)	
**Children’s physical activity (n, %)**				*p* ˂ 0.0001
Low	1459 (44.5%)	374 (51.4%)	215 (63.1%)	
Moderate	1384 (42.2%)	251 (34.5%)	84 (24.6%)	
High	438 (13.3%)	103 (14.1%)	42 (12.3%)	
**Children’s perceived stress (n, %)**				*p* ˂ 0.0001
No	2408 (73.4%)	490 (67.3%)	206 (60.4%)	
Yes	873 (26.6%)	238 (32.7%)	135 (39.6%)	
**Children’s sleep quality (n, %)**				*p* ˂ 0.0001
Adequate	2372 (72.3%)	582 (79.9%)	276 (80.9%)	
Inadequate	909 (27.7%)	146 (20.1%)	65 (19.1%)	

**Table 2:** Multivariate logistic regression analysis for children’s BMI.

Characteristics	Children’s BMI (Overweight/Obesity vs. Normal Weight)	
	OR * (95% CI **)	*p*-Value
Childhood age (over/below mean value)	0.95 (0.20–1.91)	*p* = 0.7032
Gender (male/female)	1.38 (0.97–1.75)	*p* = 0.0341
Nationality (Greek/other)	1.12 (0.32–1.99)	*p* = 0.8019
Type of residence (rural/urban)	1.09 (0.36–1.79)	*p* = 0.3011
Maternal educational level (low/moderate and high)	1.18 (0.64–1.72)	*p* = 0.2139
Family economic status (low/moderate and high)	1.39 (0.92–1.79)	*p* = 0.0293
Maternal age (over/below mean value)	1.03 (0.22–1.97)	*p* = 0.8282
Maternal smoking status (regular smokers/non-smokers)	1.31 (0.67–1.88)	*p* = 0.1254
Employment status (unemployed/employed)	1.29 (0.69–1.92)	*p* = 0.3004
Marital status (married/divorced)	0.95 (0.23–1.98)	*p* = 0.7081
Parity (multiparity/nulliparity)	1.41 (0.75–1.96)	*p* = 0.1202
Maternal pre-pregnancy BMI status (overweight and obesity/underweight and normal weight)	1.72 (1.03–2.45)	*p* = 0.0809
Maternal gestational weight gain (low and excessive/normal)	1.88 (1.41–2.33)	*p* = 0.0201
Childbirth weight (low and high/normal)	1.68 (1.45–2.06)	*p* = 0.0278
Kind of delivery (cesarean section/vaginal)	2.03 (1.78–2.39)	*p* = 0.0248
Exclusive breastfeeding (no/yes)	2.57 (2.24–2.78)	*p* = 0.0045
Children’s physical activity (low/moderate and high)	2.88 (2.58–3.31)	*p* = 0.0087
Children’s perceived stress (yes/no)	3.12 (2.93–3.58)	*p* = 0.0013
Children’s sleep quality (inadequate/adequate)	2.48 (2.25–2.92)	*p* = 0.0052

* Odds Ratio: OR; ** CI: Confidence Interval.

## Data Availability

The data are available upon request to the corresponding author.

## Supplementary Materials

The following supporting information can be downloaded at: https://www.mdpi.com/article/10.3390/metabo15060345/s1, Table S1. Descriptive statistics of the study population.

## References

[1] Zapata J.K., Gómez-Ambrosi J., Frühbeck G. (2025). Childhood obesity: The threatening apprentice of the adiposity empire. Rev. Endocr. Metab. Disord..

[2] Zhang Y., Yin Y., Zhang X., Ye J., Zhang J. (2022). Association of adverse childhood experiences with diabetes: A systematic review and meta-analysis. J. Diabetes Complicat..

[3] Hawton K., Shirodkar D., Siese T., Hamilton-Shield J.P., Giri D. (2025). A recent update on childhood obesity: Aetiology, treatment and complications. J. Pediatr. Endocrinol. Metab..

[4] Nittari G., Scuri S., Petrelli F., Pirillo I., di Luca N.M., Grappasonni I. (2019). Fighting obesity in children from European World Health Organization member states. Epidemiological data, medical-social aspects, and prevention programs. Clin. Ter..

[5] Spinelli A., Buoncristiano M., Nardone P., Starc G., Hejgaard T., Júlíusson P.B., Fismen A.-S., Weghuber D., Milanović S.M., García-Solano M. (2021). Thinness, overweight, and obesity in 6-to 9-year-old children from 36 countries: The World Health Organization European Childhood Obesity Surveillance Initiative—COSI 2015–2017. Obes. Rev..

[6] Hassapidou M., Tzotzas T., Makri E., Pagkalos I., Kaklamanos I., Kapantais E., Abrahamian A., Polymeris A., Tziomalos K. (2017). Prevalence and geographic variation of abdominal obesity in 7- and 9-year-old children in Greece; World Health Organization Childhood Obesity Surveillance Initiative 2010. BMC Public Health.

[7] Jacovides C., Pritsa A., Chrysafi M., Papadopoulou S.K., Kapetanou M.G., Lechouritis E., Mato M., Papadopoulou V.G., Tsourouflis G., Migdanis A. (2024). Childhood Mediterranean Diet compliance Is associated with lower incidence of childhood obesity, specific sociodemographic, and lifestyle factors: A cross-sectional study in children Aged 6–9 Years. Pediatr. Rep..

[8] Farajian P., Panagiotakos D.B., Risvas G., Karasouli K., Bountziouka V., Voutzourakis N., Zampelas A. (2013). Socio-economic and demographic determinants of childhood obesity prevalence in Greece: The GRECO (Greek Childhood Obesity) study. Public Health Nutr..

[9] Oudat Q., Messiah S.E., Ghoneum A.D. (2025). A Multi-level approach to childhood pbesity prevention and management: Lessons from Japan and the United States. Nutrients.

[10] Rankin J., Matthews L., Cobley S., Han A., Sanders R., Wiltshire H.D., Baker J.S. (2016). Psychological consequences of childhood obesity: Psychiatric comorbidity and prevention. Adolesc. Health Med. Ther..

[11] Williams J., Buoncristiano M., Nardone P., Rito A.I., Spinelli A., Hejgaard T., Kierkegaard L., Nurk E., Kunešová M., Milanović S.M. (2020). A snapshot of European children’s eating habits: Results from the fourth round of the WHO European Childhood Obesity Surveillance Initiative (COSI). Nutrients.

[12] Vallianou N.G., Kounatidis D., Tzivaki I., Zafeiri G.C.M., Rigatou A., Daskalopoulou S., Stratigou T., Karampela I., Dalamaga M. (2025). Ultra-processed foods and childhood obesity: Current evidence and perspectives. Curr. Nutr. Rep..

[13] Farrell A.H., Szatmari P., Vaillancourt T. (2024). Epidemiology of mental health challenges in children and adolescents. Pediatr. Clin. N. Am..

[14] Singh S., Roy D., Sinha K., Parveen S., Sharma G., Joshi G. (2020). Impact of COVID-19 and lockdown on mental health of children and adolescents: A narrative review with recommendations. Psychiatry Res..

[15] Deborah Omoleye D., Olubukola Abidakun O., Oluwadamilola Akinje R., Hannah Ademuyiwa O., Mofoluwaso Fasogbon B. (2024). A review of the effects of the COVID-19 pandemic on children and adolescents’ mental health. Curr. Pediatr. Rev..

[16] Meherali S., Punjani N., Louie-Poon S., Abdul Rahim K., Das J.K., Salam R.A., Lassi Z.S. (2021). Mental health of children and adolescents amidst COVID-19 and past pandemics: A rapid systematic review. Int. J. Environ. Res. Public Health.

[17] Zilidis C., Stuckler D., McKee M. (2020). Use of amenable mortality indicators to evaluate the impact of financial crisis on health system performance in Greece. Eur. J. Public Health.

[18] Zilidis C., Angelopoulos N.V. (2022). The impact of economic crisis on mortality due to mental health illnesses. J. Public Health.

[19] Marchionatti L.E., Schafer J.L., Karagiorga V.E., Balikou P., Mitropoulou A., Serdari A., Moschos G., Athanasopoulou L., Basta M., Simioni A. (2024). The mental health care system for children and adolescents in Greece: A review and structure assessment. Front. Health Serv..

[20] Davis S.L., Soistmann H.C. (2022). Child’s perceived stress: A concept analysis. J. Pediatr. Nurs..

[21] Achterberg M., Dobbelaar S., Boer O.D., Crone E.A. (2021). Perceived stress as mediator for longitudinal effects of the COVID-19 lockdown on wellbeing of parents and children. Sci. Rep..

[22] Kornienko D.S., Rudnova N.A., Veraksa A.N., Gavrilova M.N., Plotnikova V.A. (2024). Exploring the use of the perceived stress scale for children as an instrument for measuring stress among children and adolescents: A scoping review. Front. Psychol..

[23] Allison K.C., Parnarouskis L., Moore M.D., Minnick A.M. (2024). Insomnia, short sleep, and their treatments: Review of their associations with weight. Curr. Obes. Rep..

[24] El Halal C.D.S., Nunes M.L. (2019). Sleep and weight-height development. J. Pediatr..

[25] Bringmann H. (2019). Genetic sleep deprivation: Using sleep mutants to study sleep functions. EMBO Rep..

[26] Demichelis O.P., Grainger S.A., McKay K.T., Bourdaniotis X.E., Churchill E.G., Henry J.D. (2022). Sleep, stress and aggression: Meta-analyses investigating associations and causality. Neurosci. Biobehav. Rev..

[27] Bruni O., Malorgio E., Doria M., Finotti E., Spruyt K., Melegari M.G., Villa M.P., Ferri R. (2022). Changes in sleep patterns and disturbances in children and adolescents in Italy during the COVID-19 outbreak. Sleep Med..

[28] Jahrami H., BaHammam A.S., Bragazzi N.L., Saif Z., Faris M., Vitiello M.V. (2021). Sleep problems during the COVID-19 pandemic by population: A systematic review and meta-analysis. J. Clin. Sleep Med..

[29] Jahrami H.A., Alhaj O.A., Humood A.M., Alenezi A.F., Fekih-Romdhane F., AlRasheed M.M., Saif Z.Q., Bragazzi N.L., Pandi-Perumal S.R., BaHammam A.S. (2022). Sleep disturbances during the COVID-19 pandemic: A systematic review, meta-analysis, and meta-regression. Sleep Med. Rev..

[30] Sharma M., Aggarwal S., Madaan P., Saini L., Bhutani M. (2021). Impact of COVID-19 pandemic on sleep in children and adolescents: A systematic review and meta-analysis. Sleep Med..

[31] Kalmbach D.A., Anderson J.R., Drake C.L. (2018). The impact of stress on sleep: Pathogenic sleep reactivity as a vulnerability to insomnia and circadian disorders. J. Sleep Res..

[32] Ordway M.R., Condon E.M., Basile Ibrahim B., Abel E.A., Funaro M.C., Batten J., Sadler L.S., Redeker N.S. (2021). A systematic review of the association between sleep health and stress biomarkers in children. Sleep Med. Rev..

[33] Xu Y., Hua J., Wang J., Shen Y. (2023). Sleep duration is associated with metabolic syndrome in adolescents and children: A systematic review and meta-analysis. J. Clin. Sleep Med..

[34] Mentzelou M., Papadopoulou S.K., Papandreou D., Spanoudaki M., Dakanalis A., Vasios G.K., Voulgaridou G., Pavlidou E., Mantzorou M., Giaginis C. (2023). Evaluating the Relationship between circadian rhythms and sleep, metabolic and cardiovascular disorders: Current clinical evidence in human studies. Metabolites.

[35] Barbouzas A.E., Malli F., Daniil Z., Gourgoulianis K. (2022). Long-Term Impact of COVID-19 Pandemic in sleep quality and lifestyle in young adults. Int. J. Environ. Res. Public Health.

[36] Piątkowska-Chmiel I., Krawiec P., Ziętara K.J., Pawłowski P., Samardakiewicz M., Pac-Kożuchowska E., Herbet M. (2023). The impact of chronic stress related to COVID-19 on eating behaviors and the risk of obesity in children and adolescents. Nutrients.

[37] Hertiš Petek T., Marčun Varda N. (2024). Childhood cardiovascular health, obesity, and some related disorders: Insights into chronic inflammation and oxidative stress. Int. J. Mol. Sci..

[38] Avogaro A. (2024). Diabetes and obesity: The role of stress in the development of cancer. Endocrine.

[39] Kappes C., Stein R., Körner A., Merkenschlager A., Kiess W. (2023). Stress, stress reduction and obesity in childhood and adolescence. Horm. Res. Paediatr..

[40] Figorilli M., Velluzzi F., Redolfi S. (2025). Obesity and sleep disorders: A bidirectional relationship. Nutr. Metab. Cardiovasc. Dis..

[41] Akhlaghi M., Kohanmoo A. (2023). Sleep deprivation in development of obesity, effects on appetite regulation, energy metabolism, and dietary choices. Nutr. Res. Rev..

[42] McCoy T., Sochan A.J., Spaeth A.M. (2024). The relationship between sleep and physical activity by age, race, and gender. Rev. Cardiovasc. Med..

[43] Alenezi M.A., Alabdulathim S., Alhejaili S.A.M., Al Sheif Z.A.A., Aldossari K.K., Bakhsh J.I., Alharbi F.M., Ahmad A.A.Y., Aloufi R.M., Mushaeb H. (2024). The association between obesity and the development and severity of obstructive sleep apnea: A systematic review. Cureus.

[44] Chaput J.P., McHill A.W., Cox R.C., Broussard J.L., Dutil C., da Costa B.G.G., Sampasa-Kanyinga H., Wright K.P. (2023). The role of insufficient sleep and circadian misalignment in obesity. Nat. Rev. Endocrinol..

[45] Meneses-Echavez J.F., Iglesias-Gonzalez L.E., Loaiza-Betancur A.F., Guapo N.C. (2025). Sedentary behavior and sleep for children and adolescents with obesity: A systematic review. Ann. N. Y. Acad. Sci..

[46] Mantzorou M., Papandreou D., Vasios G.K., Pavlidou E., Antasouras G., Psara E., Taha Z., Poulios E., Giaginis C. (2022). Exclusive breastfeeding for at least four months is associated with a lower prevalence of overweight and obesity in mothers and their children after 2–5 years from delivery. Nutrients.

[47] Papandreou D., Mantzorou M., Tyrovolas S., Pavlidou E., Antasouras G., Psara E., Poulios E., Vasios G.K., Giaginis C. (2022). Pre-pregnancy excess weight association with maternal sociodemographic, anthropometric and lifestyle factors and maternal perinatal outcomes. Nutrients.

[48] Gilmore L.A., Redman L.M. (2015). Weight gain in pregnancy and application of the 2009 IOM guidelines: Toward a uniform approach. Obesity.

[49] Bacci S., Bartolucci F., Chiavarini M., Minelli L., Pieroni L. (2014). Differences in birthweight outcomes: A longitudinal study based on siblings. Int. J. Environ. Res. Public Health.

[50] World Health Organization (2006). The World Health Report: 2006: Working Together for Health. World Health Organization. https://apps.who.int/iris/handle/10665/43432.

[51] Jamesm W.P. (2008). WHO recognition of the global obesity epidemic. Int. J. Obes..

[52] Mantzorou M., Papandreou D., Pavlidou E., Papadopoulou S.K., Tolia M., Mentzelou M., Poutsidi A., Antasouras G., Vasios G.K., Giaginis C. (2023). Maternal gestational diabetes is associated with high risk of childhood overweight and obesity: A cross-sectional study in pre-school children aged 2–5 years. Medicina.

[53] Antasouras G., Papadopoulou S.K., Alexatou O., Papandreou D., Mentzelou M., Migdanis A., Psara E., Migdanis I., Chrysafi M., Tyrovolas S. (2023). Adherence to the Mediterranean diet during pregnancy: Associations with sociodemographic and anthropometric parameters, perinatal outcomes, and breastfeeding practices. Medicina.

[54] Craig C.L., Marshall A.L., Sjostrom M., Bauman A.E., Booth M.L., Ainsworth B.E., Pratt M., Ekelund U., Yngve A., Sallis J.F. (2003). International physical activity questionnaire: 12-country reliability and validity. Med. Sci. Sports Exerc..

[55] Cohen S., Kamarck T., Mermelstein R. (1983). A global measure of perceived stress. J. Health Soc. Behav..

[56] Ribeiro Santiago P.H., Nielsen T., Smithers L.G., Roberts R., Jamieson L. (2020). Measuring stress in Australia: Validation of the Perceived Stress Scale (PSS-14) in a national sample. Health Qual. Life Outcomes.

[57] Salahuddin M., Maru T.T., Kumalo A., Pandi-Perumal S.R., Bahammam A.S., Manzar M.D. (2017). Validation of the Pittsburgh Sleep Quality Index in community dwelling Ethiopian adults. Health Qual. Life Outcomes.

[58] Park S.E., So W.Y., Kang Y.S., Yang J.H. (2023). Relationship between perceived stress, obesity, and hypertension in Korean adults and older adults. Healthcare.

[59] Roy S.K., Jahan K., Alam N., Rois R., Ferdaus A., Israt S., Karim M.R. (2021). Perceived stress, eating behavior, and overweight and obesity among urban adolescents. J. Health Popul. Nutr..

[60] Richardson A.S., Arsenault J.E., Cates S.C., Muth M.K. (2015). Perceived stress, unhealthy eating behaviors, and severe obesity in low-income women. Nutr. J..

[61] Barrington W.E., Ceballos R.M., Bishop S.K., McGregor B.A., Beresford S.A. (2012). Perceived stress, behavior, and body mass index among adults participating in a worksite obesity prevention program, Seattle, 2005–2007. Prev. Chronic Dis..

[62] Farag N.H., Moore W.E., Lovallo W.R., Mills P.J., Khandrika S., Eichner J.E. (2008). Hypothalamic-pituitary-adrenal axis function: Relative contributions of perceived stress and obesity in women. J. Womens Health.

[63] Baskind M.J., Taveras E.M., Gerber M.W., Fiechtner L., Horan C., Sharifi M. (2019). Parent-perceived stress and its association with children’s weight and obesity-related behaviors. Prev. Chronic Dis..

[64] Tenk J., Mátrai P., Hegyi P., Rostás I., Garami A., Szabó I., Hartmann P., Pétervári E., Czopf L., Hussain A. (2018). Perceived stress correlates with visceral obesity and lipid parameters of the metabolic syndrome: A systematic review and meta-analysis. Psychoneuroendocrinology.

[65] Dakanalis A., Voulgaridou G., Alexatou O., Papadopoulou S.K., Jacovides C., Pritsa A., Chrysafi M., Papacosta E., Kapetanou M.G., Tsourouflis G. (2024). Overweight and obesity Is associated with higher risk of perceived stress and poor sleep quality in young adults. Medicina.

[66] Elizabeth B., Wanda D., Apriyanti E. (2021). The correlation between sleep quality and the prevalence of obesity in school-age children. J. Public Health Res..

[67] Turel O., Romashkin A., Morrison K.M. (2017). A model linking video gaming, sleep quality, sweet drinks consumption and obesity among children and youth. Clin. Obes..

[68] Yıldız F., Tuğrul Aksakal M.Z., Yıldız R., Baş F. (2024). The relationship between sleep quality, sleep duration, social jet lag and obesity in adolescents. J. Clin. Res. Pediatr. Endocrinol..

[69] Massoudi M., Pourghassem Gargari B., Asghari Jafarabadi M., Norouzi S. (2023). Major dietary patterns and sleep quality in relation to overweight/obesity among school children: A case-control study. Health Promot. Perspect..

[70] Anam M.R., Akter S., Hossain F., Bonny S.Q., Akter J., Zhang C., Rahman M.M., Mian M.A.B. (2022). Association of sleep duration and sleep quality with overweight/obesity among adolescents of Bangladesh: A multilevel analysis. BMC Public Health.

[71] Hur S., Oh B., Kim H., Kwon O. (2021). Associations of diet quality and sleep quality with obesity. Nutrients.

[72] Doo M., Wang C. (2020). Associations among Sleep Quality, Changes in eating habits, and overweight or obesity after studying abroad among international students in South Korea. Nutrients.

[73] Hawes N.J., Wiggins A.T., Reed D.B., Hardin-Fanning F. (2019). Poor sleep quality is associated with obesity and depression in farmers. Public Health Nurs..

[74] Kanellopoulou A., Notara V., Magriplis E., Antonogeorgos G., Rojas-Gil A.P., Kornilaki E.N., Lagiou A., Yannakoulia M., Panagiotakos D.B. (2021). Sleeping patterns and childhood obesity: An epidemiological study in 1,728 children in Greece. J. Clin. Sleep Med..

[75] Flegal K.M., Kruszon-Moran D., Carroll M.D., Fryar C.D., Ogden C.L. (2016). Trends in obesity among adults in the United States, 2005 to 2014. JAMA..

[76] Salahuddin M., Pérez A., Ranjit N., Kelder S.H., Barlow S.E., Pont S.J., Butte N.F., Hoelscher D.M. (2017). Predictors of severe obesity in low-income, predominantly Hispanic/Latino children: The Texas childhood obesity research demonstration study. Prev. Chronic Dis..

[77] Segal A.B., Huerta M.C., Aurino E., Sassi F. (2021). The impact of childhood obesity on human capital in high-income countries: A systematic review. Obes. Rev..

[78] Pavlidou E., Papadopoulou S.K., Alexatou O., Tsourouflis G., Antasouras G., Louka A., Chatziprodromidou I.P., Mentzelou M., Sampani A., Chrysafi M. (2023). Association of gestational hypertension with sociodemographic and anthropometric factors, perinatal outcomes, breastfeeding practices, and Mediterranean diet adherence: A cross-sectional study. Medicina.

[79] Hernández-Barrera L., Trejo-Valdivia B., Téllez-Rojo M.M., Baccarelli A., Wright R., Cantoral A., Barquera S. (2024). Pre-gestational obesity and gestational weight gain as predictors of childhood obesity. Arch. Med. Res..

[80] Papadopoulou S.K., Mentzelou M., Pavlidou E., Vasios G.K., Spanoudaki M., Antasouras G., Sampani A., Psara E., Voulgaridou G., Tsourouflis G. (2023). Caesarean section delivery is associated with childhood overweight and obesity, low childbirth weight and postnatal complications: A cross-sectional Study. Medicina.

[81] Gorman T., Maher G.M., Al Khalaf S., Khashan A.S. (2024). The association between caesarean section delivery and obesity at age 17 years. Evidence from a longitudinal cohort study in the United Kingdom. PLoS ONE.

[82] Sulley I., Saaka M. (2022). Relationship between caesarean section delivery and risk of overweight/obesity among children aged 6–23 months in the Tamale Metropolis of Ghana. J. Nutr. Sci..

[83] Li W., Yuan J., Wang L., Qiao Y., Liu E., Wang S., Leng J. (2022). The association between breastfeeding and childhood obesity/underweight: A population-based birth cohort study with repeated measured data. Int. Breastfeed. J..

[84] Pojednic R., D’Arpino E., Halliday I., Bantham A. (2022). The benefits of physical activity for people with obesity, independent of weight loss: A systematic review. Int. J. Environ. Res. Public Health.

[85] Almonacid-Fierro A., González-Almonacid J. (2022). The pandemic of childhood obesity: Challenges and possibilities from physical activity. Health Promot. Perspect..

[86] Hidding L.M., Chinapaw M.J.M., van Poppel M.N.M., Mokkink L.B., Altenburg T.M. (2018). An updated systematic review of childhood physical activity questionnaires. Sports Med..

