# Supplementary material for: Childhood Obesity and Overweight Are Associated with Higher Risk of Perceived Stress and Poor Sleep Quality: A Cross-Sectional Study in Children Aged 6–9 Years
Source: Metabolites. 2025 May 22;15(6):345. doi: 10.3390/metabo15060345 (PMC12195024; doi:10.3390/metabo15060345)
Supplement: Supplementary file 1 [file metabolites-15-00345-s001.zip › metabolites-3629821-supplementary.pdf]

Table S1. Descriptive statistics of the study population

Characteristics (n=4350)	Descriptive statistics
**Childhood age (mean±SD; years)**	7.5±1.2
**Gender (n, %)**	
Male	2166 (49.8%)
Female	2184 (50.2%)
**Nationality (n, %)**	
Greek	4164 (95.7%)
Other	186 (4.3%)
**Type of residence (n, %)**	
Urban	2854 (65.6%)
Rural	1496 (34.4%)
**Maternal educational level (n, %)**	
Low	1316 (30.3%)
Moderate	1854 (42.6%)

High	1180 (27.1%)
**Family economic status (n, %)**	
Low	1827 (42.0%)
Moderate	1631 (37.5%)
High	892 (20.5%)
**Maternal age (mean±SD; years)**	35.4±5.1
**Maternal smoking habits (n, %)**	
No smokers	3227 (74.2%)
Regular smokers	1123 (25.8%)
**Employment status (n, %)**	
Employed	2979 (68.5%)
Unemployed	1371 (31.5%)
**Marital status (n, %)**	
Married	2968 (68.2%)
Divorced	1382 (31.8%)

**Parity (n, %)**	
Nulliparity	2815 (64.7%)
Multiparity	1535 (35.3%)
**Maternal pre-pregnancy BMI status (n, %)**	
Underweight	126 (2.9%)
Normal weight	3285 (75.5%)
Overweight	741 (17.0%)
Obese	198 (4.6%)
**Maternal gestational weight gain (n, %)**	
Low	641 (14.7%)
Normal	2022 (46.5%)
Excessive	1687 (38.8%)
**Childbirth weight (n, %)**	
Low (< 2500gr)	344 (7.9%)
Normal (2500-4000gr)	3533 (81.7%)

High (> 4000gr)	453 (10.4%)
**Kind of delivery (n, %)**	
Vaginal	1900 (43.7%)
Caesarean section	2450 (56.3%)
**Exclusive breastfeeding (n, %)**	
No	2167 (49.8%)
Yes	2183 (50.2%)
**Children physical activity (n, %)**	
Low	2048 (47.1%)
Moderate	1719 (39.5%)
High	583 (13.4%)
**Children perceived stress (n, %)**	
Low	2704 (62.2%)
Moderate	1417 (32.6%)
High	229 (5.2%)

**Children sleep quality (n, %)**	
No adequate	1120 (25.8%)
Adequate	3230 (74.2%)
